# In vivo performance of Al_2_O_3_-Ti bone implants in the rat femur

**DOI:** 10.1186/s13018-021-02226-7

**Published:** 2021-01-22

**Authors:** Marjan Bahraminasab, Samaneh Arab, Manouchehr Safari, Athar Talebi, Fatemeh Kavakebian, Nesa Doostmohammadi

**Affiliations:** 1 Nervous System Stem Cells Research Center, Semnan University of Medical Sciences, Semnan, Iran; 2 Department of Tissue Engineering and Applied Cell Sciences, School of Medicine, Semnan University of Medical Sciences, Semnan, Iran; 3 Faculty of Metallurgical and Materials Engineering, Semnan University, Semnan, Iran

**Keywords:** In vivo, Composites, Bone formation, Orthopedic biomaterials, Osteocytes

## Abstract

**Background:**

Alumina-titanium (Al_2_O_3_-Ti) biocomposites have been recently developed with improved mechanical properties for use in heavily loaded orthopedic sites. Their biological performance, however, has not been investigated yet.

**Methods:**

The aim of the present study was to evaluate the in vivo biological interaction of Al_2_O_3_-Ti. Spark plasma sintering (SPS) was used to fabricate Al_2_O_3_-Ti composites with 25 vol.%, 50 vol.%, and 75 vol.% Ti content. Pure alumina and titanium were also fabricated by the same procedure for comparison. The fabricated composite disks were cut into small bars and implanted into medullary canals of rat femurs. The histological analysis and scanning electron microscopy (SEM) observation were carried out to determine the bone formation ability of these materials and to evaluate the bone-implant interfaces.

**Results:**

The histological observation showed the formation of osteoblast, osteocytes with lacuna, bone with lamellar structures, and blood vessels indicating that the healing and remodeling of the bone, and vasculature reconstruction occurred after 4 and 8 weeks of implantation. However, superior bone formation and maturation were obtained after 8 weeks. SEM images also showed stronger interfaces at week 8. There were differences between the composites in percentages of bone area (TB%) and the number of osteocytes. The 50Ti composite showed higher TB% at week 4, while 25Ti and 75Ti represented higher TB% at week 8. All the composites showed a higher number of osteocytes compared to 100Ti, particularly 75Ti.

**Conclusions:**

The fabricated composites have the potential to be used in load-bearing orthopedic applications.

## Introduction

Pure titanium (Ti) is a well-established material in orthopedic implants, due to the favorable combination of properties including biocompatibility, mechanical strength, and chemical stability [1, 2]. Despite this, Ti requires improvement of mechanical properties if it is intended to be used in heavily loaded sites [3, 4]. To make Ti suitable for load-bearing applications, its alloys and composites have been extensively developed using materials such as tantalum, zirconium, niobium, molybdenum, magnesium, and calcium phosphates, to name a few [5, 6].

Composites are hybrid materials which consist of two or more distinct materials (on a macroscopic scale) selectively designed to provide superior properties rather than its constituents alone [7, 8]. A composite differs from an alloy in which the main constituent is a metal combined with one or more other elements and retains all the properties of a metal in the resulting material (such as electrical conductivity, and ductility). Ceramic-metal composites are one of the most successful groups which can favorably combine the dissimilar properties of ceramic and metal constituents in one material system. Ceramic materials, such as alumina (Al_2_O_3_), cause negligible osteolysis because of its low friction coefficient and reduced wear debris generation [9–14]. This, along with high hardness, favorable compressive strength, and chemical stability in physiological environments, has made it the material of choice for making orthopedic prostheses (such as alumina-on-alumina hip implants). Therefore, adding alumina into Ti can solve the low wear resistance and particle detachment from Ti implants. On the other hand, alumina alone is inherently brittle and susceptible to fracture. Therefore, the combination of alumina with Ti as a toughening material can solve this problem too.

Al_2_O_3_-Ti biocomposites were fabricated by spark plasma sintering (SPS), in the last decade, and their mechanical and corrosion characteristics were evaluated which showed promising results [15–20]. Superior flaw tolerance and toughness were obtained for 25 vol.% Ti rather than pure Al_2_O_3_ [16]. Moreover, several Al_2_O_3_-Ti composites with different metal weight percentages were made [17] and the changes in mechanical properties (elastic modulus and hardness) were studied. It was found that the elastic modulus and the fracture toughness were respectively increased and decreased by the increase in Al_2_O_3_ percentage in the composites. In addition, Fujii et al. [18] evaluated the influence of different raw powders (pure Ti and TiH_2_) on the mechanical behavior of Al_2_O_3_-Ti composites. They found higher elastic modulus, bending strength, and hardness of the composites made by TiH_2_, but lower fracture toughness compared with using pure Ti. Moreover, Bahraminasab et al. [15] fabricated a five-layer functionally graded material and uniform composites of Al_2_O_3_-Ti with 25-75 vol.% Ti, and found good mechanical properties including high hardness.

Despite the favorable properties, before any clinical practice, the biological performance of these materials must be thoroughly examined. To the best of the authors’ knowledge, there is no in vivo study available to assess the material-tissue interaction of these composites. The only in vitro study is that of Guzman et al. [21] in which a composite having 25 vol.% Ti was investigated for cytotoxicity, and its influence on cell proliferation, differentiation, and adhesion.

The aim of the present study, therefore, was to preliminarily examine the effects of these new composites on biological performance in an animal model.

## Materials and methods

### Sample preparation

**Fig. 1:**
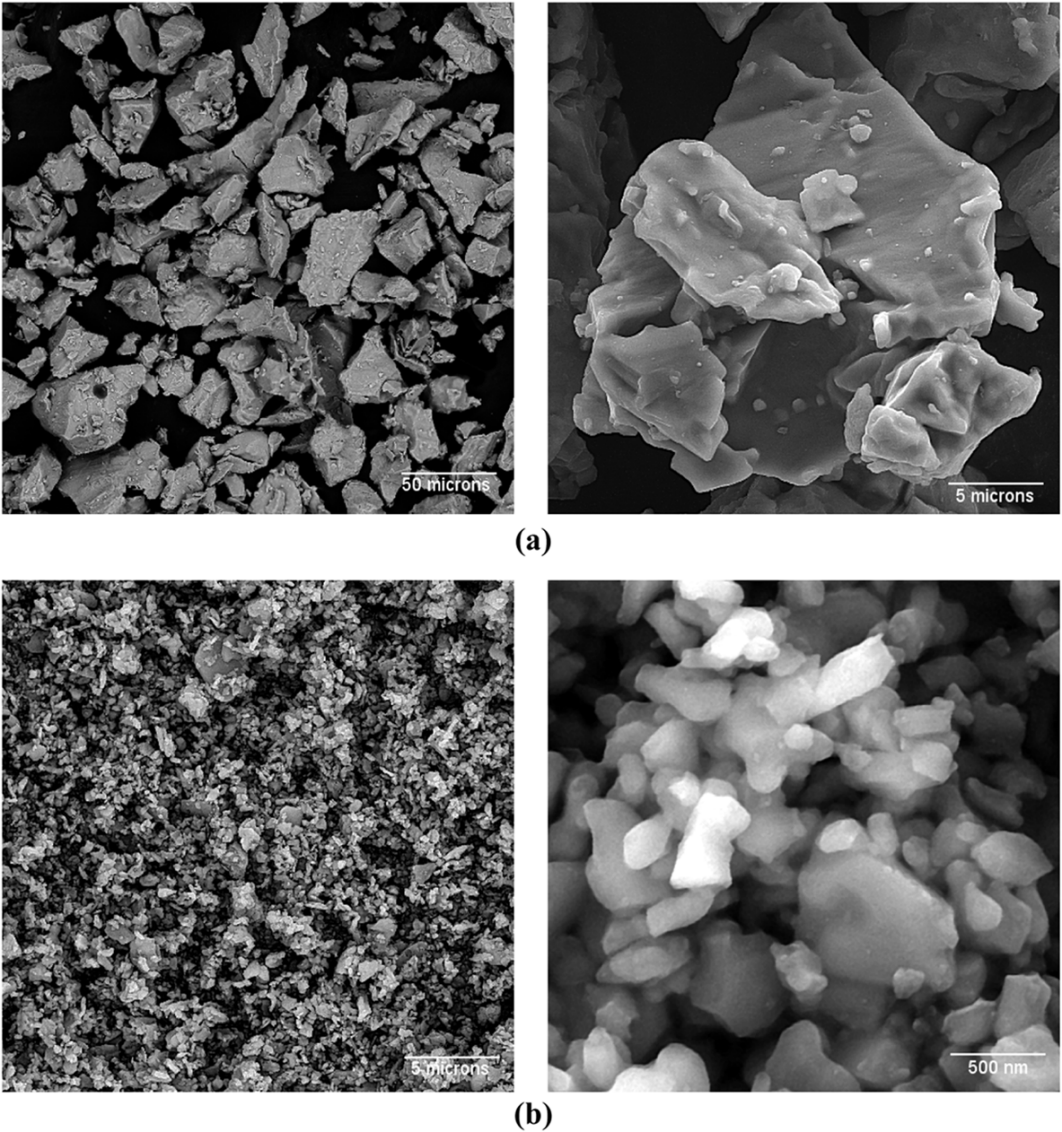
SEM images of the powders used for making composites; (**a**) Ti and (**b**) Al_2_O_3_

**Fig. 2:**
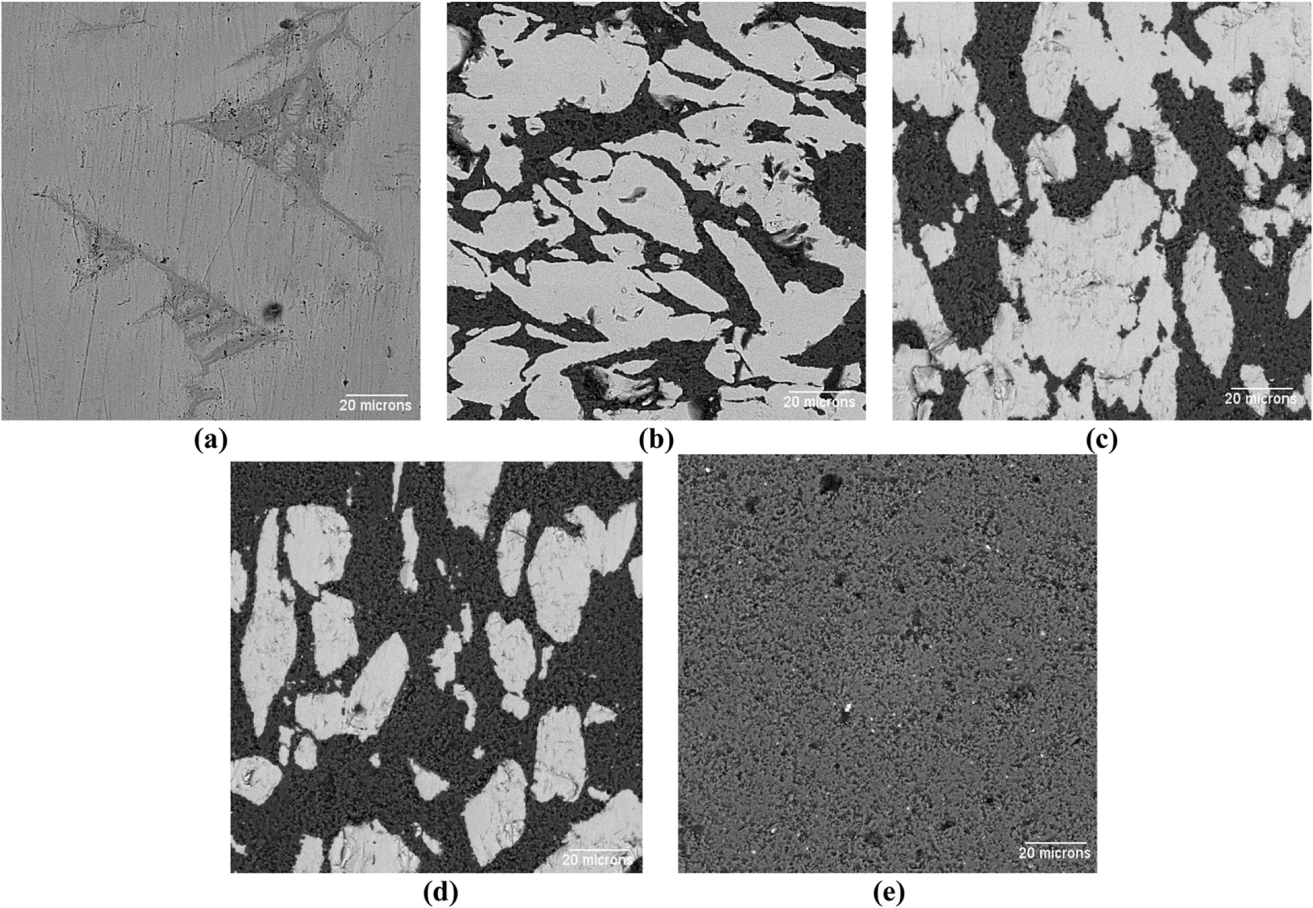
SEM images of the fabricated composites before implantation; (**a**) 100Ti, (**b**) 75Ti, (**c**) 50Ti, (**d**) 25Ti, and (**e**) 0Ti (the bright regions are Ti phase, and the dark regions are Al_2_O_3_ phase)

The samples were made using the SPS machine (SPS-20 T-10, Easy Fashion Metal Products Trade CO. Ltd. China) according to our previous study [15]. Briefly, the α-Al_2_O_3_ and Ti powders were used as the starting materials. The powders were weighed and mixed to make mixtures with different vol.% based on Table 1. The powder mixtures were then ball milled (PM400, Retsch, Germany) to grind and provide homogenized mixture. To do this, zirconia jar and balls were employed at a speed of 100 rpm for 1 h, with a ball to powder ratio of 5:1. Subsequently, the homogenized powders were added into a graphite die and sintered by the SPS machine. Figures 1 and 2 show the SEM images of the starting powders and the fabricated samples, respectively. As it can be seen in Fig. 1, the particle size used was different; coarse Ti particles vs fine Al_2_O_3_ powder. It has been indicated that fine titanium particles used for fabrication of Al_2_O_3_-Ti composites causes formation of a thin oxide layer which covers the particles [17]. This oxide layer disturbs the sintering of the powder mixture and induces the formation of uncontrolled and undesirable phases at the Al_2_O_3_-Ti interface. Therefore, in this study, larger Ti particles were used to avoid this problem, similar to previous studies [15–17, 21]. Furthermore, the use of polydisperse size distributions is effective where the small particles within the powder mixture fill the voids between the larger particles providing full density parts.

**Table 1:** Materials compositions, codes, and process parameters

Groups	Abbreviated names	Compositions	Ball mill conditions	SPS conditions			
				Applied pressure (MPa)	Maintaining time at applied pressure (min)	Sintering temperature (°C)	Soaking time (min)
1	0Ti	100 vol.% Al_2_O_3_ powder	Ball to powder ratio: 5:1 Rotating speed: 100 rpm Time: 1 h	10 20 40	3 10 Until the end	1350	30
2	25Ti	25 vol.% Ti powder-75 vol.% Al_2_O_3_ powder					3
3	50Ti	50 vol.% Ti powder-50 vol.% Al_2_O_3_ powder					3
4	75Ti	75 vol.% Ti powder-25 vol.% Al_2_O_3_ powder					3
5	100Ti	100 vol.% Ti powder					3

**Fig. 3:**
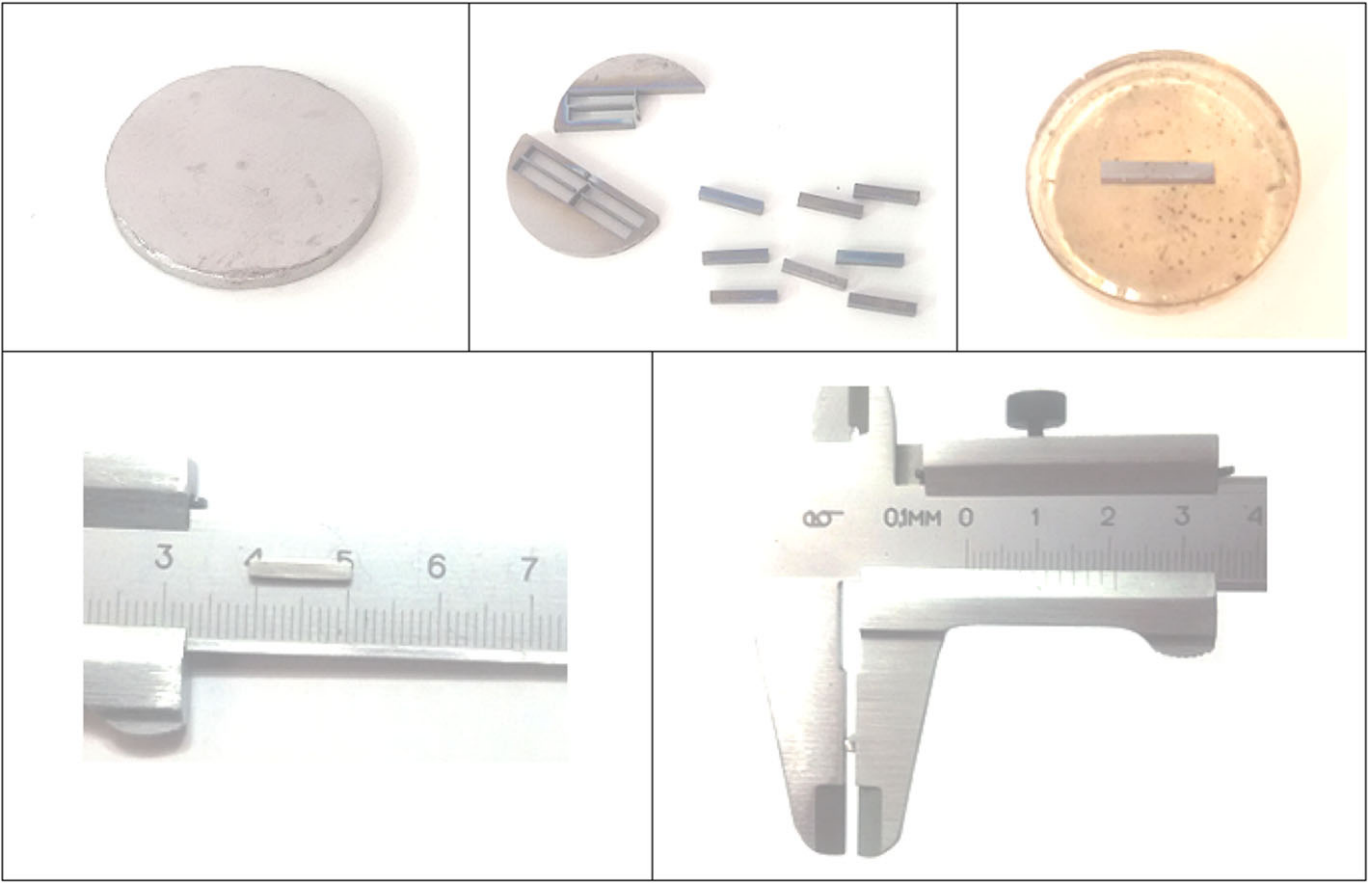
Samples prepared from 100Ti for implantation in rat femur

After fabrication, the samples were grinded to remove the graphite from their surfaces. 100Ti, 75Ti, and 50Ti were wire-cut, and 25Ti and 0Ti were cut by a micro-cutter to provide small pieces for implantation. These were successively mounted and grinded to provide bar-shaped samples with a mean size of 1.9 (± 0.08) × 1.7 (± 0.15) × 10.8 (± 0.73) mm^3^. The mounts were removed and the samples were finally grinded on all sides up to 2000-grit (Fig. 3). Before implantation, the prepared bars were ultrasonically cleaned with alcohol and distilled water, respectively, each for 10 min. The samples were then covered by aluminum foil, and sterilized by heating within a hot air oven at 100 °C for 1 h. The sterilized samples were then immediately transferred to a laminar flow bench (class II) and kept there without opening the foil cover until the time of surgery.

### Implantation

**Fig. 4:**
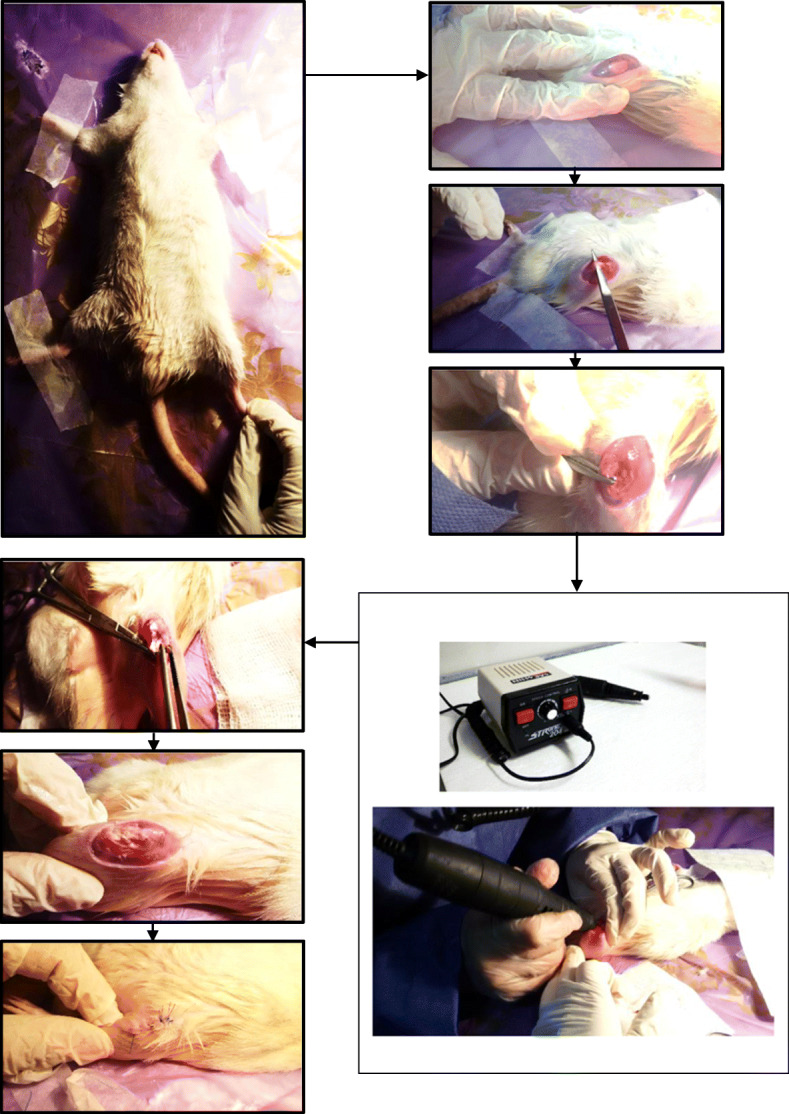
The steps in operation from anesthesia to joint closing with sutures

All animal protocols were approved by the research council and ethics committee of the authors’ affiliated institutions. Forty same-aged (16 weeks) male Wistar rats with a mean weight of 305 g (± 16 g) were selected based on a previous study [22]. The adult rats were used here, as the peri-implant bone volume and osseointegration have shown to be greater rather than the young male rats [23]. General anesthesia was done by intraperitoneal (IP) injection of a combination of ketamine hydrochloride and xylazine hydrochloride with the volume ratio of 8:2 at 1 cc/kg. The surgical procedure was carried out under sterile conditions. The skin covering the knee joint area was shaved and cleaned with 70% alcohol pads. An incision of about 2.5 cm was made on the knee of the left hind leg. The intercondylar space of the knee joint was exposed by making an incision of 1–1.5 cm on the lateral side of the patella and lifting the patella and its ligament to the medial side. A hole was drilled between the knee condyles into the bone marrow cavity which was subsequently widened and drilled lengthwise to provide a hollow passageway matching the implant size. The drilling was done under profuse physiological saline to keep the joint surface wet and avoid necrosis due to the heat generated through drilling. At the end, the debris such as bone fragments was rinsed out by physiological saline using a syringe. A sample was then placed in the femur hollow passageway and pushed deep enough to avoid the implant tip to disturb the joint surface. The patella and its ligament were pulled back laterally to its initial place and the skin was closed with sterile sutures (Fig. 4). No postoperative antibiotic treatment and pain-killer was given. The rats were monitored after surgery and it was seen that they used the operated leg naturally. The implanted animals were kept to a housing facility, where they could access to water and food, freely.

### Radiographic and weight analyses

The radiographic images were taken immediately after the surgery and before sacrificing, to ensure whether the implants were appropriately placed in situ and whether they had no movements. Furthermore, the rats were weighed every week until the end of implantation time.

### Histological analysis

After 4 and 8 weeks, the rats were sacrificed and their femurs were carefully dissected and collected. The bones were then fixed in 10% neutral-buffered formalin, and decalcified in 10% formic acid for 2-3 weeks. During the decalcification process, the femurs were monitored and after a few days when the bones became somewhat rubbery, the implants were carefully removed and the bones were allowed to fully decalcify. Afterward, the standard dehydration was performed in serially increasing ethanol solutions (to 100% ethanol) which was followed by immersion of the samples in xylene, paraffin-saturated xylene, and lastly molten paraffin. Transverse sections with thickness of 5 μm were provided from the center of the implantation sites using a microtome and stained with hematoxylin and eosin (H&E). The peri-implant tissues were observed under an optical microscope. The number of osteocytes was counted in 4 regions in each image with a frame size of 500 × 300 pixels. Furthermore, the area of trabecular bone and whole tissue was measured and TB% was calculated by dividing the trabecular bone area to the whole tissue area. These measurements were conducted using the ImageJ software.

### SEM observation

Some sections of non-decalcified bones were prepared for scanning electron microscopy (SEM). The bone samples were fixed in formalin and dehydrated in increasing concentrations of ethanol. They were then put into the plastic cylindrical molds which were filled by epoxy resin and cut into several pieces transversely using a micro-cutter.

### Statistical analysis

Statistical analyses were conducted through analysis of variance (ANOVA) using the Minitab V17 software with the confidence level of 95% (*α* = 0.05). The normal probability plot of residuals was checked in all analyses. Furthermore, the post hoc pairwise comparisons were performed using the Tukey test.

## Results

### Radiographic and weights analyses

**Fig. 5:**
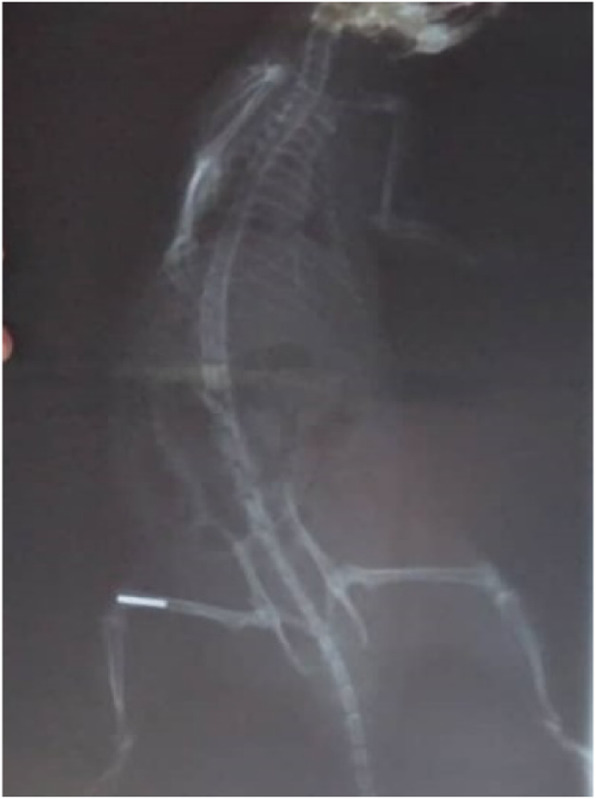
The radiographic image of a rat with 0Ti implant material after the surgery

The radiographic images exhibited that the implants were appropriately placed in situ and they had no movements post-surgery. Figure 5 shows the radiographic image taken from a rat with 0Ti material.

**Fig. 6:**
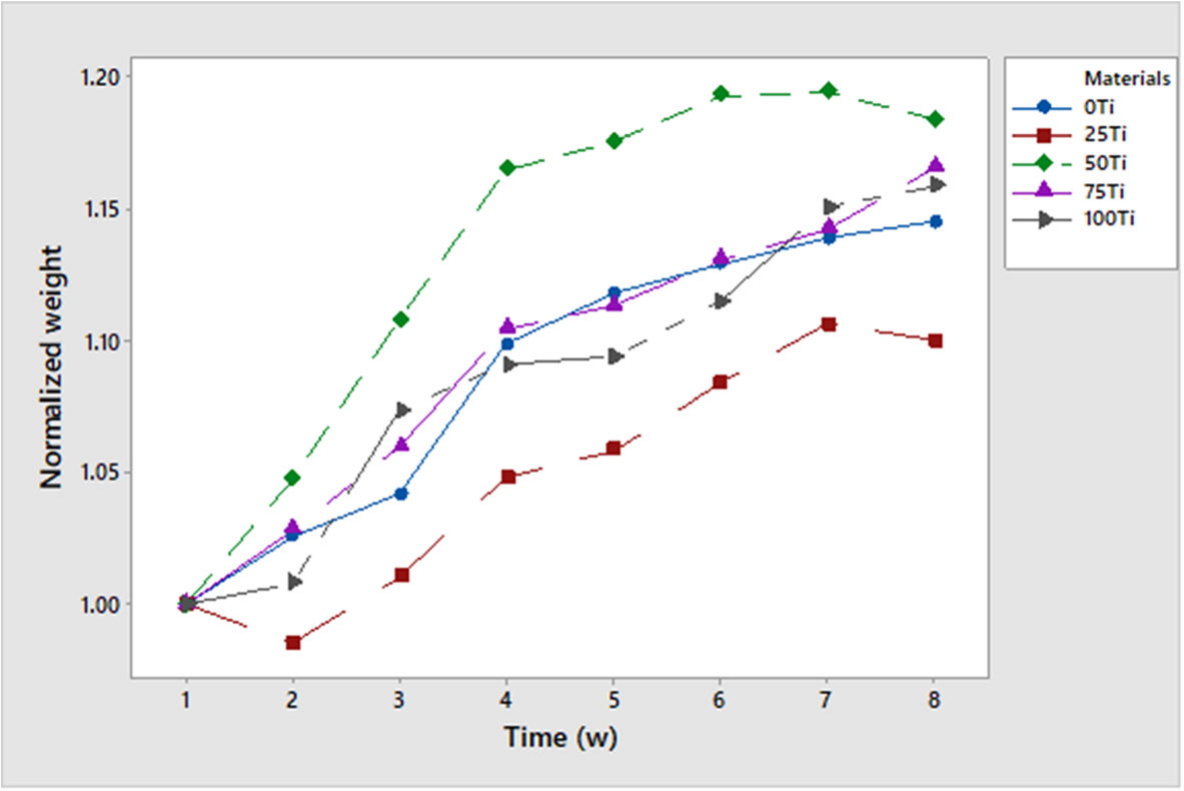
Variations of weights during 8 weeks postoperatively

Figure 6 shows the variations in rats’ weights for 8 weeks postoperatively. As it can be seen, the rats in all groups had their routine weight gain. Only the rats with 25Ti implant had a slight weight loss at the beginning but they could gain weight in a similar trend as the other groups. In this figure, the means of normalized weights are presented, which were calculated by dividing the weight obtained in each week by the weight measured in the first week, at each group.

### Histological and SEM observation

**Fig. 7:**
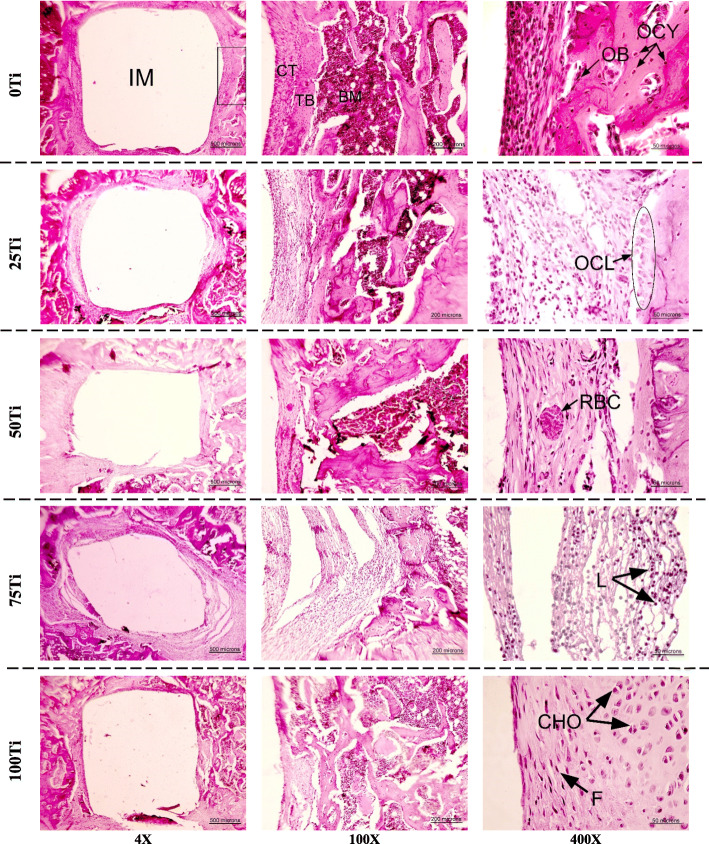
H&E images in different groups after 4 weeks. IM, implant site; TB, trabecular bone; BM, bone marrow; CT, connective tissue; OCY, osteocytes; OB, osteoblast lining cells; OCL, osteoclast; RBC, red blood cell; L, lymphocytes; CHO, chondrocytes; F, fibroblasts

**Fig. 8:**
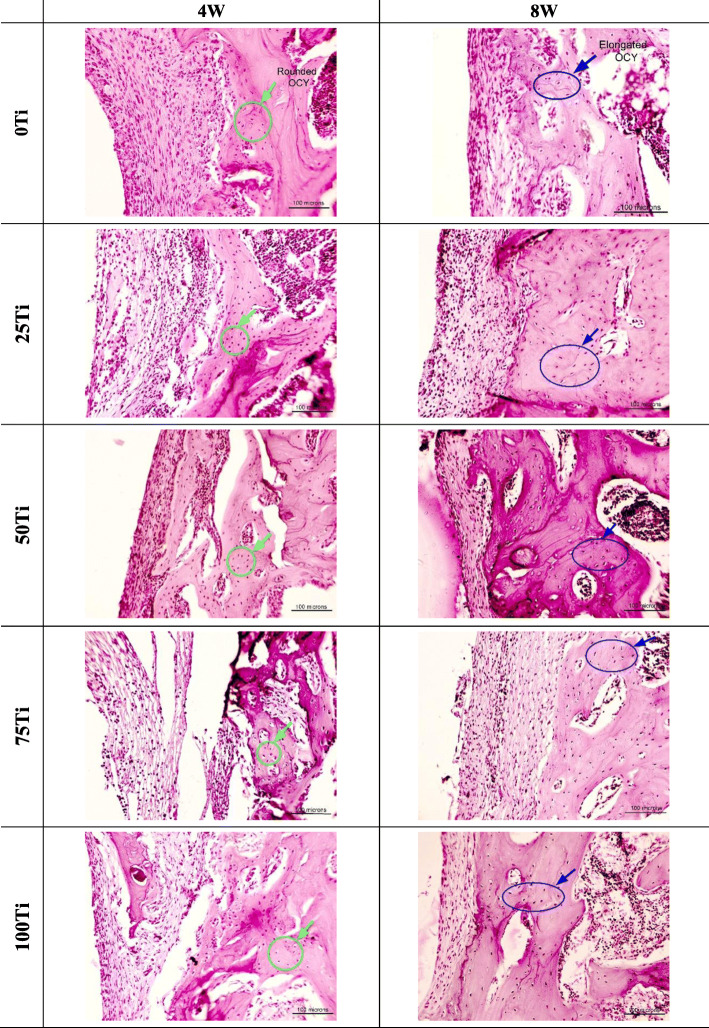
Comparison of H&E sections of peri-implant tissue in different groups at two-time points. Magnification, × 200

The histological observation showed no inflammation or other implant-associated complications macroscopically for all implant materials. In microscopic scale, however, a large number of lymphocytes was observed only in the histopathology sections provided from the peri-implant tissue of 75Ti implant after 4 weeks (Fig. 7, row 4). The lymphocytes, however, were not observed after 8 weeks of implantation. The connective tissue and new bone were seen around the implants in all groups. As it can be clearly seen in Fig. 7, the connective tissue around the 75Ti implant was very loose and fragile after 4 weeks which became stronger and firmer after 8 weeks. Nevertheless, the connective tissues in all groups had similar morphology 8 weeks after implantation. The progenitor cells and fibroblasts were observed in the connective tissues of all groups. However, there were differences between the connective tissues around different materials after 4 and 8 weeks (Fig. 8). The connective tissues after 8 weeks of implantation were firmer with no macrophages and giant cells meaning that no debris of implanted materials were in the surrounding tissues to activate these cells for phagocytosis. The morphology of osteocytes was also slightly different at weeks 4 and 8 (Fig. 8); more rounded versus elongated morphology (green circles vs blue ovals). Moreover, the formation of osteoblast, osteocytes with lacuna, bone with lamellar structures, and blood vessels indicated that the healing and remodeling of the bone, and vasculature reconstruction occurred after 4 and 8 weeks of implantation.

**Fig. 9:**
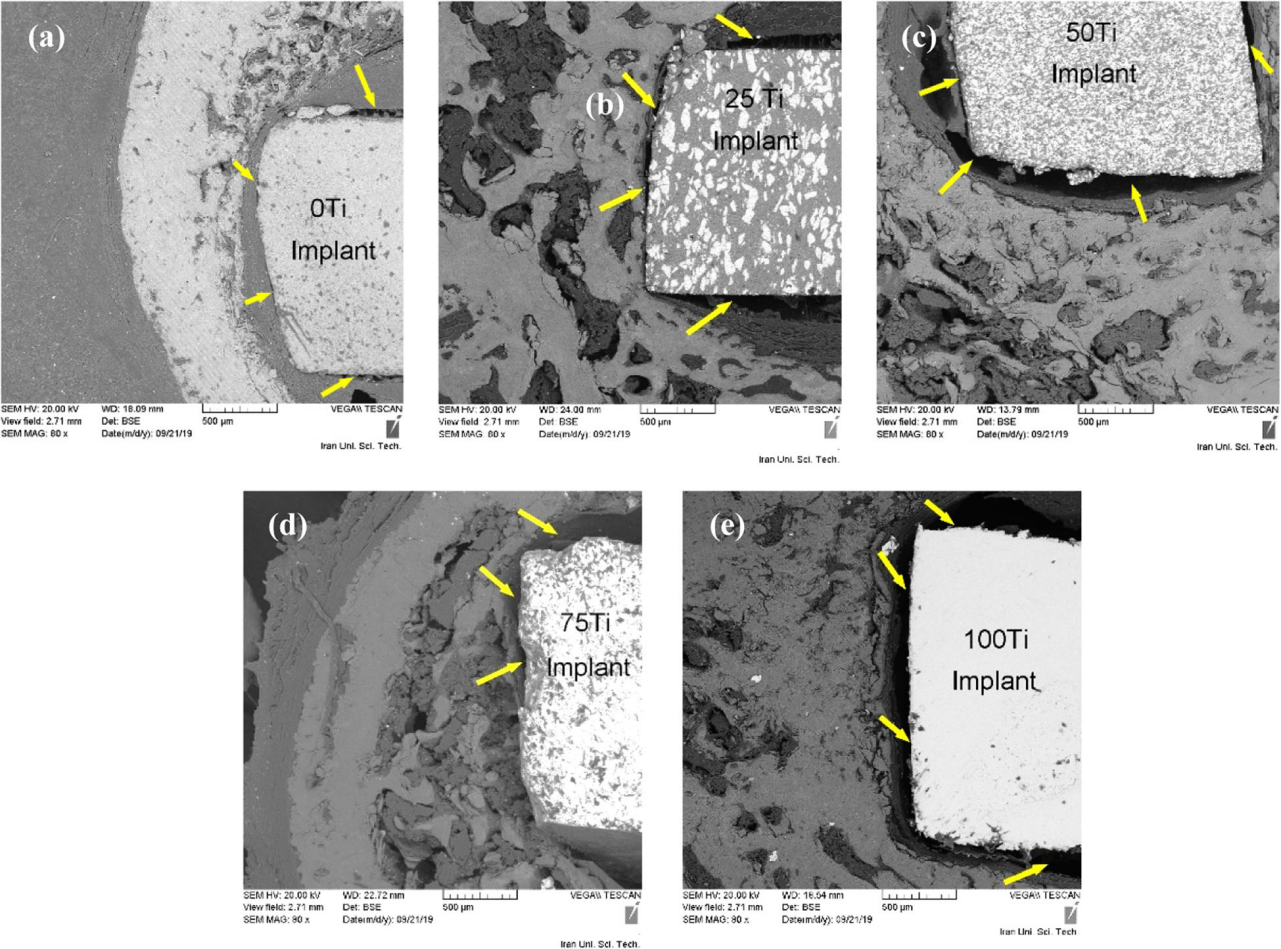
SEM images of implant materials and peri-implant tissues after 4 weeks

**Fig. 10:**
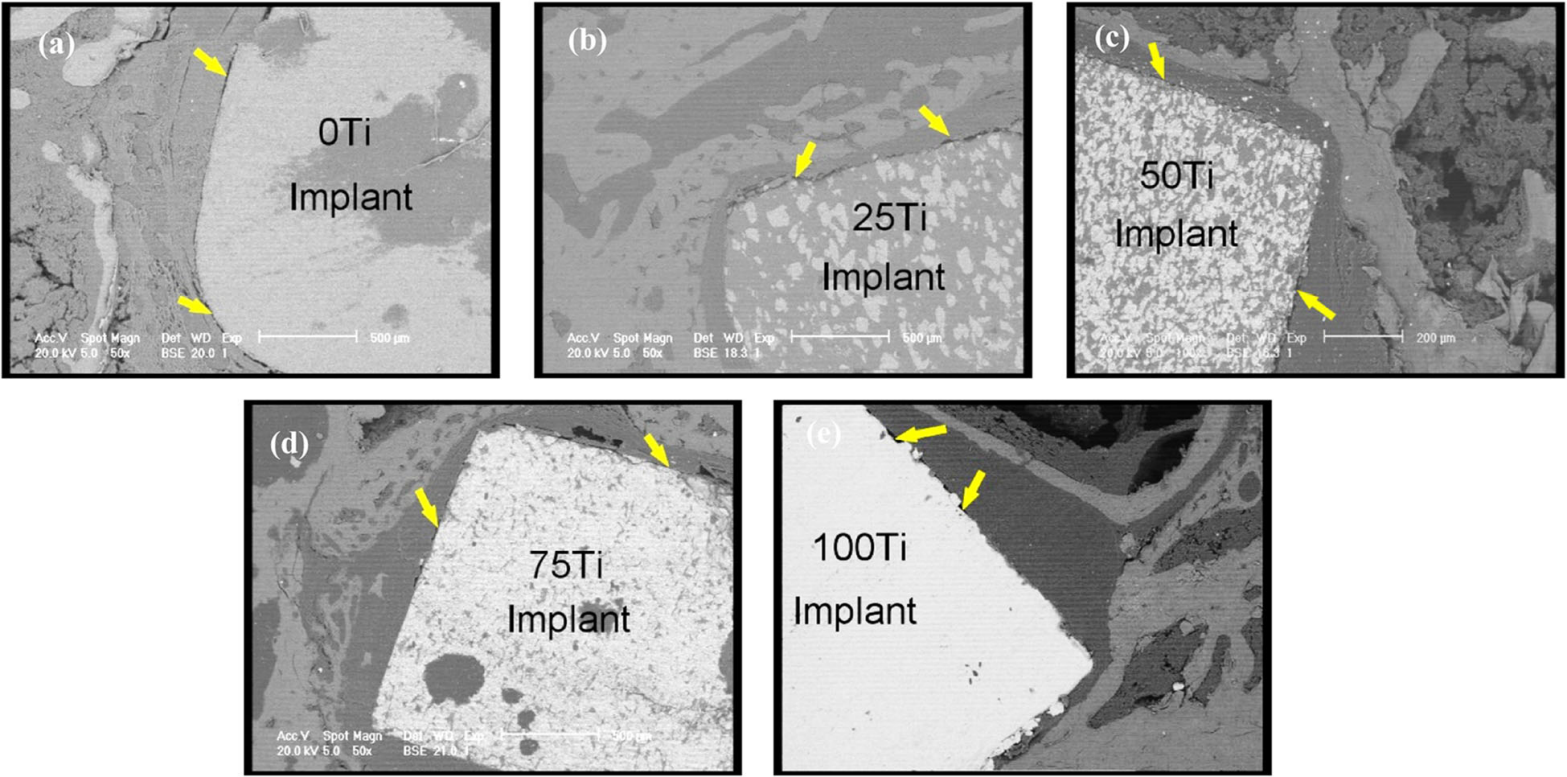
SEM images of implant materials and peri-implant tissues after 8 weeks

SEM images in Fig. 9 revealed the existence of gaps at the implant-bone interfaces meaning that the implant-tissue interfaces 4 weeks after surgery were not strong enough (yellow arrows). This is very obvious for 75 Ti implant material (group 4) which is in agreement with the histological results obtained here. However, after 8 weeks, the bonds between the implants and the peri-implant tissues became stronger and there were no gaps in some regions and very narrow and small gaps in some other parts of the interface (Fig. 10, yellow arrows).

**Fig. 11:**
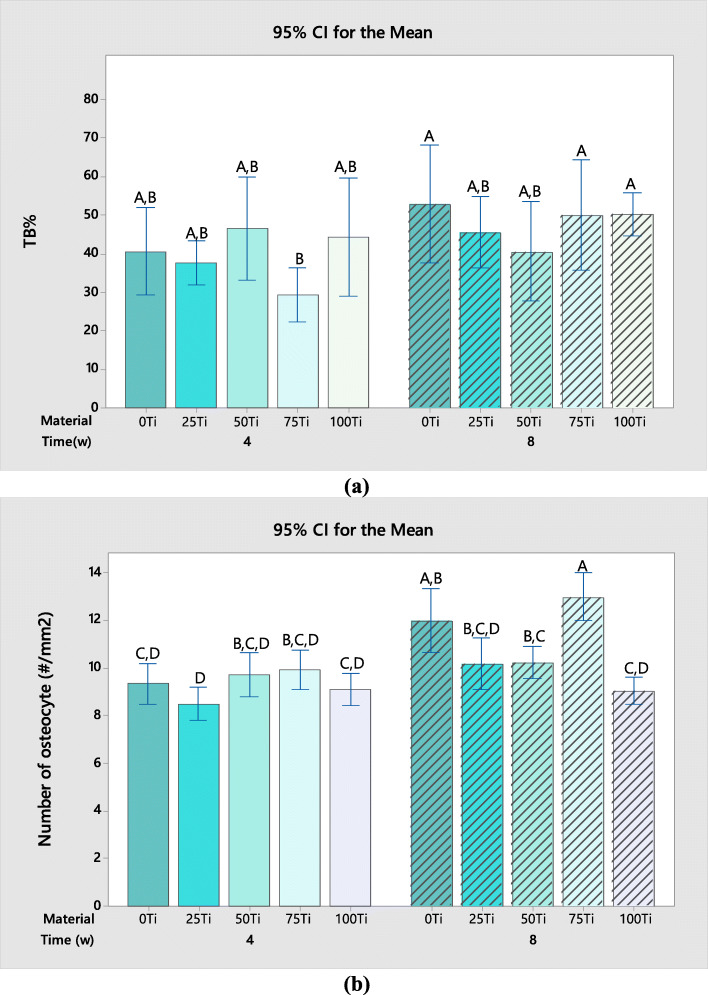
Comparison of (**a**) TB%, and (**b**) number of osteocytes at different groups; the means that do not share a letter are significantly different

Two-way ANOVA on the TB% around the materials showed that the only significant factor was time (*P* value = 0.006), meaning the time period following the operation influenced the bone formation. The TB% increased in all groups by time except for 25Ti for which the TB% was decreased from 46.64 to 40.61% (Fig. 11a). The most increase in TB% was found to be 20.99% for 75Ti followed by 0Ti, 25Ti, and 100Ti with 12.36%, 7.99%, and 5.9% increase, respectively. The interesting point is that despite the inflammatory response (presence of lymphocytes) observed in 75Ti at week 4, the bone formation was very high in the next 4 weeks. The Tukey pairwise comparisons between the groups are also shown in Fig. 11a, in which the means that do not share a letter are significantly different. Therefore, there were no significant differences between the TB% of the implants at week 4 and also at week 8. It was shown that the TB% around 0Ti, 75Ti, and 100Ti after 8 weeks was significantly higher than that of 75Ti after 4 weeks.

Furthermore, two-way ANOVA was employed to evaluate the number of osteocytes in the adjacent bones around the biomaterials. Since the data did not have a normal distribution, the statistical analysis was performed on the transformed data [24]. The material and time were found to be significant factors (*P* values of 0.000) meaning that both the postoperative time and the composition of studied materials influenced the number of osteocytes. Furthermore, the interaction effect of material and time was also statistically significant (*P* value = 0.003). As it can be seen in Fig. 11b, the number of osteocytes increased by time in all groups except for 100Ti for which a negligible decrease was found (from 9.11 to 9.04#/mm^2^). The increase in the number of osteocytes along with variation of morphology from round to elongated shape (as indicated in H&E stained sections) means the maturation of the new bone by time. The results of Tukey test are also shown in Fig. 11b, by letters above the bars. There were no significant differences between the numbers of osteocytes at week 4. Furthermore, it was indicated that the number of osteocytes in the bone around 75Ti after 8 weeks (mean value of 12.99#/mm^2^) was significantly higher than those of other materials. The osteocyte density around 0Ti after 8 weeks was also significantly higher than 0Ti, 25Ti, and 100Ti after 4 weeks, and 100Ti after 8 weeks. The lowest number of osteocytes was observed in the bone around 25Ti at week 4.

## Discussion

Al_2_O_3_-Ti composites are promising materials for load-bearing orthopedic implants due to high mechanical properties. The assessment of their biological performances, in the present study, offers preliminary evidence that these composites provide bone formation in vivo. Our findings showed the formation of connective tissue and new bone around all the implant materials. The multipotent stem cells and progenitor cells in the connective tissue may contribute to the formation of trabecular bone within the medullary canal [25]. The only material with fibrous tissue, early (4 weeks) inflammatory response, and low formation of new bone was 75Ti. However, at late (8 weeks), the normal tissue growth was found for 75Ti even superior to other composites meaning that the conversion of fragile fibrous tissue into cartilage and finally bone tissue occurred. Usually, a localized inflammatory reaction occurs after implantation of an orthopedic device in response to the invasive surgery and to the presence of the prosthesis [26]. All materials placed into the living tissues induce a host immune response which reflects the initial tissue repair steps. The first contact of a biomaterial with tissue causes adsorption of proteins from blood and interstitial fluids on its surface within a few nanoseconds. This protein layer is determinant for the activation of a complex series of precisely controlled responses including coagulation cascade, complement system, platelets, and immune cells [27]. This results in the initiation of the inflammatory response. What is important is to avoid its continuation that leads to chronic inflammation, foreign body reactions, and consequently the loss of the required function [28]. The results obtained here 8 weeks after the operation did not show any chronic inflammatory response even for 75Ti implant material. Furthermore, the formation of osteoblast lining cells, osteocytes with lacuna, trabecular and lamellar bone structures, and blood vessels are indicative of the bone healing, remodeling, and vasculature reconstruction after 4 and 8 weeks of implantation.

The time had a positive effect on TB% around 0Ti, 25Ti, 75Ti, and 100Ti, and on the number of osteocytes in all groups except for 100Ti. Previous studies on Ti implant also demonstrated that an increase in postoperative time increases the bone area around the implants [29–31]. For example, Ballo et al. [31] evaluated the bone tissue response to 4 implant materials including strontium- and silicon- substituted apatite (Sr-HA and Si-HA) modified titanium implants, pure HA, and pure Ti. Their results indicated that the bone area formed around all implants including Ti increased by time. This is in agreement with the results obtained here. A higher osteocyte density adjacent to the surfaces of implants can possibly be associated with the recruitment of higher osteoprogenitor cells resulting in a higher number of osteoblasts becoming embedded within the extracellular matrix (ECM) which finally differentiate into osteocytes [32]. It has been indicated that the time of implantation influences the number of osteocytes in peri-implant bone [33]. Another factor that affects the number of osteocytes is the mechanical loading because the osteocytes act as mechanosensors and regulate mineralization [34]. In peri-implant bone tissue, these cells physically communicate with the surface of implant material through canaliculi and respond to the mechanical loading [35]. Different materials transfer the loads differently to the adjacent tissues due to a material property known as elastic modulus/stiffness [36]. Therefore, the different numbers of osteocytes can be expected for different materials. One point that should be noted is that the surgical procedure also plays an important role in the number of osteocytes and the healing time. It has been indicated that the drilling speed and generated heat during the preparation of the implant site causes osteocyte apoptosis leading to extensive remodeling and delayed healing [37, 38]. Furthermore, the primary mechanical stability and the contact stresses at the bone-implant interface are also important in avoiding the implant micromotion, and fibrous membrane formation which subsequently affects the osseointegration [39]. To control these, in the present study, all operations were conducted by a single person with the same drill and drilling speed, and saline serum was rinsed to the implantation site during the drilling to lessen the effect of the generated heat. Meanwhile, the prepared holes were manually filled with implants as tightly as possible to provide initial mechanical stability.

In hard tissue replacement, where the implant material interfaces with the bone such as non-cemented components of the knee and hip prostheses, new bone formation and anchorage of the implant with bone are required. The biocompatibility of an implanted material is ideal if the material provokes the normal tissue formation at its surface and creates a connected interface that can transfer the loads normally experienced at the implantation site. In this study, the implant-bone interface in all groups became stronger with very small gaps at week 8, as indicated in SEM images. Several studies measured the shear strength/modulus of the bone bond with different implant materials through the push-out test and obtained higher bond strength by the time. For example, Huang et al. [40] tested the in vivo performance of two implant materials in a rabbit model. Their push-out test showed higher shear strength for both materials at week 12 compared to week 4. Moreover, Bandyopadhyay et al. [41] conducted push-out test on dense Ti, porous Ti, and porous Ti with carbon nano-tube implanted in rat femur in two-time points, and indicated the increase in shear modulus by time in all materials. The SEM observation in their study, revealed distinct gaps at the interface of tissue-implant in all materials after 4 weeks, particularly for dense Ti. However, the gaps at the interfaces were reduced after 10 weeks. Similar observations were obtained in our SEM images.

One point that should be emphasized is that some metals used in metallic implants such as nickel, titanium, and aluminum are nonessential for human health. These metals when present at sufficiently high concentrations can disturb normal biological functions and result in cellular stress responses known as metal toxicity [42]. Metal toxicity exerts influence on various tissues including the kidney, liver, heart, and nervous systems [43, 44]. Adverse effects of Al have been repeatedly discussed in several studies. Neurodegenerative diseases such as Alzheimer’s as well as certain bone diseases and dialysis dementia have been attributed to Al exposure [45]. There is evidence that exposure to Al may lead to increased oxidative stress, inflammatory events, and the breakdown of the blood-brain barrier (BBB) [43]. Also, with the widespread use of titanium, there are concerns regarding the adverse effects of titanium accumulation and its effects on the human body [44, 46]. For example, increased titanium levels were noted in the lungs, spleen, muscle, and regional lymph nodes in implanted animals [47, 48]. The fabricated composites here are intended to be used in load-bearing applications such as total joint replacements; therefore, Al_2_O_3_ wear debris may produce during articulation which can induce biological responses. One study evaluated the effects of sub-acute exposure to Al_2_O_3_ nanoparticles on oxidative stress and histological changes in mouse liver and brain by oral route exposure for 21 days [44]. The findings showed that exposure to these particles produced oxidative stress in erythrocyte, liver, and brain. Furthermore, the neurotoxicity of the nanoparticles appeared to be possible due to increase in dopamine and norepinephrine levels in the brain cerebral cortex and increased brain oxidative stress. Moreover, another in vivo study on rats (intranasally instilled with nano-Al_2_O_3_ once per day for 15–30 days) demonstrated that exposure to these nanoparticles can be a risk factor for neurodegenerative diseases and might negatively influence the hippocampus, striatum, and dopaminergic neurons [49]. However, in vitro study on the effect of metal oxide nanoparticles including Al_2_O_3_ on Neuro-2A cells showed that Al_2_O_3_ had no measurable effect on the cells until the concentrations reached greater than 200 μg/mL (LDH leakage and change in mitochondria activity) [50]. Another study [51] compared the cytotoxicity of clinically relevant Co-Cr alloy and Al_2_O_3_ ceramic wear particles. Their results showed that Co-Cr particles 50 and 5 μm^3^ per cell reduced the viability of U937 cells by 97% and 42% and reduced the viability of L929 cells by 95% and 73%, respectively. However, only, at 50 μm^3^ per cell, the Al_2_O_3_ ceramic particles reduced U937 cell viability by 18%. In our histological analysis, however, no material debris was found in the peri-prosthetic tissues and the materials were very biostable. Nevertheless, it is believed that further analyses are required to examine the concentration and accumulation of Ti and Al_2_O_3_ in the biological fluids and organs after long time use of the composites in load-bearing sites.

The properties of Al_2_O_3_-Ti composites are mostly favorable for load-bearing orthopedic implants. The mechanical strength of orthopedic implants is a prime factor to sustain the loads of daily activity. High hardness (or low coefficient of friction) is desired to avoid wear and the subsequent osteolysis; however, it should be accompanied by acceptable fracture toughness to avoid brittle failure. High corrosion resistance is also favorable to minimize corrosion and ion release to the surrounding tissues. Another important factor is the elastic modulus (or stiffness) of a material which can contribute to an undesirable phenomenon known as the stress shielding effect. It is related to changes in load transfer and attributed to large differences between the relatively high elastic modulus of the material and the adjoining bone. Currently used materials in total joint replacements including Co-Cr alloy, stainless steel, Ti alloys, alumina, and zirconia all have much higher elastic moduli (about 240, 210, 112, 350, and 200 GPa, respectively) than that of bone (≤ 30 GPa) [13]. The fabricated composites here have elastic moduli between those of Ti and Al_2_O_3_ which are still much higher. One way to reduce elastic modulus is the use of functionally graded/hierarchical materials with a harder and stiffer material at the articular surface and low modulus material at the bone interface to simultaneously reduce wear and stress shielding [52]. However, this requires advance manufacturing technologies such as additive manufacturing/3D printing [53].

The present paper is a preliminary study on the bone formation of Al_2_O_3_-Ti composites in vivo. It would be beneficial that more in-depth analyses on the morphological features of the trabecular bone are conducted in the future research to better understand the osteogenesis process around these implant materials. One way to achieve a faster healing process is the surface modifications of the materials and the use of bioactive coatings. It would be interesting to investigate the effect of surface treatment on the biological response of the fabricated composites in future work.

## Conclusion

In vivo performance of Al_2_O_3_-Ti bone implants was assessed by implantation in the rat femur. Among the fabricated composites, 50Ti showed superior early bone formation, while 25Ti and 75Ti provided higher bone formation at late (8 weeks). The tissue response of these composites was superior or comparable to pure Ti implant (100Ti) which is the benchmark material. Therefore, they can be promising for clinical applications.

## Data Availability

The data used and/or analyzed in the present study are available from the corresponding author upon reasonable request.

## Abbreviations

SPS: Spark plasma sintering
SEM: Scanning electron microscope
IP: Intraperitoneal
H&E: Hematoxylin and eosin
ANOVA: Analysis of variance
